# Skin Lesions in Diabetes Mellitus: A 6-Year Observational Study and 11-Year Longitudinal Analysis of Hospital Admissions from NE Romania

**DOI:** 10.3390/life16060957

**Published:** 2026-06-05

**Authors:** Madalina Marinescu, Gina E. Botnariu, Mădălina Mocanu, Dan Vâță, Doinița Temelie-Olinici, Ioana Halip, Adriana-Ionela Patrascu, Ioana A. Popescu, Dragoș F. Gheuca-Solovastru, Laura Gheuca-Solovastru

**Affiliations:** 1 Discipline of Dermatology-Venereology, Department of Medical Sciences III, Faculty of Medicine “Grigore T. Popa”, University of Medicine and Pharmacy, 700115 Iași, Romania; dr.madalinamarinescu@gmail.com (M.M.); dan.vata@umfiasi.ro (D.V.);; 2 Clinic of Dermatology-Venereology, “Sf. Spiridon” Emergency County Clinical Hospital, 700111 Iași, Romania

**Keywords:** diabetes mellitus, skin lesions, xerosis, pruritus, predictive markers, retrospective study

## Abstract

Chronic hyperglycemia profoundly impairs skin integrity, with dermatological complications affecting up to half of patients with diabetes mellitus. The objective of this study was to describe the spectrum, frequency, and clinical characteristics of skin lesions in patients with diabetes mellitus and to evaluate whether specific cutaneous signs are associated with an increased risk of subsequently developing type 2 diabetes. This retrospective observational study evaluated 960 cases admitted to the Dermatology Clinic of “Sf. Spiridon” Emergency County Clinical Hospital, Iași, between 2017 and 2022, complemented by an 11-year longitudinal follow-up (2011–2016). Ulcerative lesions predominated (85.7%), followed by inflammatory manifestations (12.4%), while classical diabetes-specific dermatoses represented <2% of cases. Poor glycemic control (HbA1c > 7%) was documented in 94.2% of patients, with the most severe lesions occurring in those with HbA1c > 10%. In the longitudinal analysis, patients who later developed diabetes initially presented significantly higher rates of xerosis, pruritus, and callus compared with the controls. Multivariate logistic regression identified xerosis (OR 4.70) and pruritus (OR 3.41) as independent predictors of future diabetes. These findings suggest that certain dermatological signs may serve as early non-invasive markers of metabolic dysfunction and highlight the importance of routine skin examination in diabetes risk stratification.

## 1. Introduction

Diabetes mellitus is a heterogeneous metabolic disease characterized primarily by chronic hyperglycemia, insulin resistance, and disturbances of protein and lipid metabolism, which progressively affect multiple organs and reduce patients’ quality of life [1]. Cutaneous manifestations are common, being reported in up to 50% of individuals with diabetes, and may occur either as direct consequences of metabolic dysregulation or secondary to microvascular, neuropathic, and immune dysfunction [2]. Experimental findings indicate impaired keratinocyte proliferation and differentiation, reduced synthesis of free fatty acids and cholesterol, increased oxidative stress, and altered collagen remodeling, all of which weaken the skin barrier and delay healing [3].

Clinically, patients frequently experience decreased pressure sensitivity, diminished protective reflexes, xerosis, pruritus, hyperkeratosis, and callus formation. Neuropathy and microangiopathy are central contributors to these changes, promoting tissue hypoxia, poor resistance to mechanical stress, and susceptibility to ulceration. Neuropathic ulcers are typically painless, may develop granulation tissue, and are prone to superinfection, often resulting in prolonged hospitalization and, in severe cases, amputation [4]. Poor glycemic control also increases the frequency of recurrent bacterial and fungal infections, including onychomycosis, balanoposthitis, oropharyngeal candidiasis, and interdigital intertrigo, which facilitate bacterial entry and further risk of ulceration [4]. The most frequently implicated pathogens in skin infections among diabetes patients are Staphylococcus aureus and β-hemolytic streptococci [5].

Because dermatological manifestations may precede or accompany diabetes, they can serve as clinically relevant indicators of disease status and metabolic imbalance. Early recognition and adequate management of skin changes require coordinated care integrating clinical monitoring, patient education, self-management training, and psychosocial support, all of which are essential components of modern diabetes standards of care [6,7,8]. Structured educational interventions have demonstrated benefits in improving treatment adherence, glycemic control, patient satisfaction, and overall quality of life [9,10]. Therefore, systematic dermatological evaluation should be considered part of comprehensive diabetes management, with the potential to prevent complications and reduce hospitalization rates [11,12,13].

Previous studies have reported that cutaneous manifestations occur in approximately 30–90% of individuals with diabetes, although prevalence varies depending on population, study design, and diagnostic criteria [14]. The most common conditions include xerosis, which affects up to 50% of patients, pruritus (10–25%), onychomycosis (15–40%), and bacterial skin infections, particularly those caused by *Staphylococcus aureus* and β-hemolytic streptococci [15,16]. Neuropathic and vascular complications contribute to the development of foot ulcerations, which have a lifetime incidence of approximately 15–25% in diabetes and often require multidisciplinary management [6]. These data highlight both the high frequency and clinical relevance of dermatological involvement in diabetes and reinforce the need for systematic dermatologic assessment in this patient population.

The present study aimed to describe the spectrum and characteristics of dermatological conditions in patients with diabetes mellitus admitted to a regional specialized hospital and to examine their distribution according to demographic and clinical factors, including glycemic control. A secondary objective was to evaluate whether certain dermatological patterns were associated with repeated admissions and could serve as early indicators of increased complication risk.

## 2. Materials and Methods

### 2.1. Study Design

This was a single-center retrospective observational study including patients with diabetes mellitus admitted for the diagnosis and treatment of dermatological conditions at the “St. Spiridon” Dermatology Clinic in Iași, the largest tertiary referral center in Northeastern Romania. The primary study period covered the years 2017–2022. An extended comparative analysis was additionally conducted on a broader time interval (2011–2016) to assess patterns of repeated hospitalizations. For this secondary analysis, 60 patients with diabetes and 60 patients without diabetes (control group) were selected based on documented prior admissions for dermatological conditions. Ethics Approval: The study was conducted in accordance with the Declaration of Helsinki and received approval from the Ethics Committee of the “Grigore T. Popa” University of Medicine and Pharmacy, Iași (Approval No. 274/22.02.2023), and the Ethics Committee of the “Sf. Spiridon” Emergency County Clinical Hospital, Iași (Approval No. 119/28.12.2022).

### 2.2. Patients

#### Selection Criteria

Patients were eligible for inclusion if they met the following conditions:

(1) Age ≥ 18 years;
(2) Primary admission to the dermatology clinic for the diagnosis and/or treatment of a dermatological condition;
(3) Previously established diagnosis of type 1 or type 2 diabetes mellitus (for the main study cohort), or no diagnosis of diabetes at the time of the first admission (for the comparative longitudinal analysis).

No additional exclusion criteria were applied, as the purpose of the study was to reflect real-world clinical presentations in a tertiary dermatology service.

Patients under 18 years of age were excluded because pediatric diabetes and dermatological conditions follow distinct clinical, metabolic, and therapeutic patterns compared with adults. Our study focused on adult patients admitted to a tertiary dermatology service, where the overwhelming majority of admissions involve adults with type 2 diabetes or long-standing type 1 diabetes. Additionally, pediatric dermatology and diabetes cases are managed through dedicated pediatric services in our hospital system, and including these patients would have introduced heterogeneity in disease mechanisms and care pathways.

### 2.3. Data Collection

Data were extracted from electronic and paper-based hospitalization records. For all eligible patients, the following variables were recorded:

- Demographic characteristics (age, sex, residence: urban/rural);
- Dermatological diagnoses at admission, including diagnoses across successive admissions in patients who developed diabetes versus those who did not;
- Diabetes-related data: type of diabetes, duration of disease (when available), and glycated hemoglobin (HbA1C) levels at admission.

### 2.4. Lesion Classification

For the purpose of categorical analysis, dermatological conditions on admission were grouped into two main categories based on clinical presentation:

- Inflammatory lesions—defined as non-ulcerative, primarily erythematous or edematous skin conditions characterized by inflammation of the epidermis and/or dermis, including but not limited to dermatitis, eczema, erysipelas, cellulitis without ulceration, panniculitis, and inflammatory dermatoses such as psoriasis or lichen planus. These lesions were characterized by erythema, warmth, edema, desquamation, or pruritus without tissue loss.
- Ulcerative lesions—defined as lesions involving loss of skin integrity with partial- or full-thickness disruption of the epidermis and/or dermis, regardless of etiology. This category included neuropathic ulcers, venous ulcers, arterial ulcers, mixed vascular ulcers, pressure ulcers, traumatic ulcers, and infected or superinfected lesions. Ulcerative lesions were characterized by a visible breach in the skin surface, exudate, and variable depth.
- Lesions such as onychomycosis, callus/hyperkeratosis, xerosis, vitiligo, xanthelasma, and other non-inflammatory, non-ulcerative presentations were classified as “other lesions”.

### 2.5. Primary Versus Incidental Dermatological Findings

For each hospitalization, the primary reason for admission was identified based on the main diagnosis recorded at discharge. Skin lesions were classified as primary when they represented the reason for admission and required targeted therapeutic intervention (e.g., ulcer care, wound debridement, treatment of cellulitis, or management of superinfection).

Dermatological findings were classified as incidental when they were documented during physical examination but were not the primary cause for admission and did not require acute intervention (e.g., xerosis, callus, mild interdigital fungal infection).

### 2.6. Statistical Analysis

Data processing was performed using IBM SPSS Statistics, version 29.0 (IBM Corp., Armonk, NY, USA). Descriptive statistics included means and standard deviations for normally distributed variables and medians with interquartile ranges for non-normally distributed variables (distribution tested using the Kolmogorov–Smirnov test). Categorical variables were compared using Pearson’s χ^2^ test only when all expected cell counts were ≥5. When any expected cell count was <5, we used exact tests: the Fisher–Freeman–Halton exact test for R × C contingency tables (e.g., inflammatory vs. ulcerative vs. other across strata) and Fisher’s exact test for 2 × 2 tables. Continuous variables were compared with Student’s t-test or the Mann–Whitney U test, as appropriate. Two-sided *p* < 0.05 was considered statistically significant. To identify independent factors associated with specific dermatological manifestations, a multivariate logistic regression analysis was performed. Variables with *p* < 0.10 in univariate analysis were entered into the multivariate model. Results were expressed as odds ratios (OR) with 95% confidence intervals (CI). Statistical significance was defined as *p* < 0.05.

### 2.7. Multivariate Analysis

Variables associated with the outcome at *p* < 0.10 in univariate analysis were entered into a multivariate binary logistic regression model to identify independent predictors. The model was constructed using the Enter method. Odds ratios (ORs) with 95% confidence intervals (95% CI) were calculated. Prior to inclusion, variables were assessed for multicollinearity using variance inflation factor (VIF) values (VIF < 2 was considered acceptable). Model calibration was evaluated using the Hosmer–Lemeshow goodness-of-fit test, and model discrimination was assessed by the area under the receiver operating characteristic curve (AUC). Statistical significance was defined as *p* < 0.05.

Patient consent was waived because the study used anonymized, retrospective medical records and no identifiable data were collected.

## 3. Results

### 3.1. General Patient Characteristics and Types of Dermatological Lesions Diagnosed at Admission

The retrospective survey of the hospital records from 2017 to 2022 led to the inclusion of 960 patients with established diabetes mellitus. Of them, 51.8% were men, 57% were over the age of 65, and 58.3% were urban residents. The vast majority had type 2 diabetes (96.3%).

The classification of skin lesions in the literature was not reflected in the incidence of lesions diagnosed in these patients; thus, we decided it would be more informative to organize and analyze the cases in accordance with the reality of our clinical practice. Many types described in the literature were, in fact, rare among these diabetes patients, e.g., necrobiosis lipoidica, acanthosis nigricans, diabetic dermopathy, psoriasis, xanthoma, xanthelasma, scleroderma, vitiligo, and glucagonoma (only 18 cases in 6 years, amounting to a mere 1.9%). This is not to say that such pathologies were not seen in other patients without diabetes. Instead, diabetes patients presented mainly with ulcerative lesions (85.7%) and inflammatory lesions (12.4%).

The patients’ general characteristics relative to the types of diagnosed lesions are summarized in Table 1. Ulcerative lesions were slightly more common in men than in women (87.2% vs. 84.2%) and in rural rather than urban residents (87.2% vs. 83.9%), while more men presented inflammatory lesions (13.9% vs. 11%). Nonetheless, these differences were not statistically significant, as indicated by the *p* values of the calculated Pearson Chi-squared coefficients.

#### Primary vs. Incidental Skin Findings

Of the 960 admissions included in the study, skin pathology represented the primary reason for hospitalization in 88.1% of cases (846/960). These admissions were predominantly related to ulcerative lesions, particularly lower limb ulcers requiring local wound management, infection control, and vascular or diabetic foot evaluation.

In contrast, 11.9% of admissions (114/960) involved incidental dermatological findings, most commonly xerosis, callus formation, and mild fungal infections, which were documented during routine physical examination while patients were hospitalized for other inflammatory or non-ulcerative dermatological conditions.

This distinction reflects the greater clinical and healthcare impact of ulcerative lesions, which frequently required multidisciplinary management, compared with non-ulcerative findings, which were generally chronic, mild, and managed in an outpatient setting.

The majority of ulcerative lesions (57.2%) were located on the shanks and dorsally, while 11.4% were plantar lesions. Lower limb ulcerations were more common in patients under the age of 65 compared to older patients (57.4% vs. 54.8%). They were also more common in men compared to women (58.3% vs. 54.4%) and in patients from urban rather than rural backgrounds (59% vs. 52.6%).

In addition, venous ulcers were diagnosed in 18.6% of cases, and 16.2% of these presented evidence of bacterial infection with *Staphylococcus aureus* (14.7%) or *Beta-hemolytic Streptococci* (1.5%). A range of mycoses were also identified, mainly onychomycoses of the toenails (21.7%), and most were associated with interdigital intertrigo (20.9%), as well as balanitis, vulvovaginitis, stomatitis, and cutaneous mycoses (4.58%).

### 3.2. Incidence of Main Lesion Types Relative to the Patients’ Underlying Diabetes Condition

The glycated hemoglobin levels recorded on admission revealed poor glycemic control (HbA1C > 7%) in almost all cases (94.2%), and 89.3% of patients already had histories of unspecified, diabetes-related complications. This aspect could not be explored further because standard record-keeping procedures require admitting physicians to note only the presence or absence of complications, without details.

Ulcerative lesions were the most common regardless of the type of underlying diabetes, as can be seen in Table 2. The differences noted in the incidence of ulcerative and inflammatory lesions in patients with type 1 vs. type 2 diabetes were not statistically significant. However, patients with type 2 diabetes presented with a wider range of other lesions, while patients with type 1 diabetes did not.

In addition, deep lesions occurred in over 85% of patients with either diabetes type, while superficial lesions were much less frequent (14%). Patients with HbA1C levels in excess of 10% had the most severe and complicated lesions, e.g., superinfected ulcerations on the lower limbs.

### 3.3. Multi-Annual Incidence of Main Lesion Types in Diabetes Patients (2017–2022)

**Figure 1:**
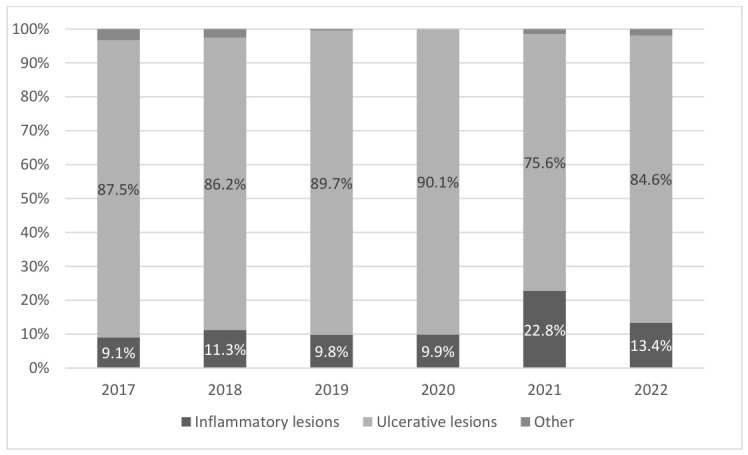
Multi-annual incidence of inflammatory, ulcerative, and other lesions (2017–2022).

The annual incidence data summarized in Table 2 and Figure 1 reveal that ulcerative lesions ranged from 75.6% (in 2021) to 90.1% (in 2020). Conversely, inflammatory lesions ranged from 9.1% (in 2017) to 22.8% (in 2021), and the differences between 2017 and 2021 were statistically significant (*p* = 0.015). Other lesion types were either absent (in 2020) or rare (3.4% in 2017). Apart from these fluctuations, this multi-annual view of admissions reveals a relatively stable situation, with ulcerative lesions being overwhelmingly common across all 6 years. The annual fluctuation and evolution of dermatological conditions over the 6-year study period are detailed in Table 3 and visually illustrated in Figure 1, demonstrating a highly stable and predominant incidence of ulcerative lesions across all analyzed years.

### 3.4. Characteristics of Patients with Previous Admissions for Dermatological Conditions

The medical records of the 960 diabetes patients included in the study were screened for evidence of previous hospitalizations in the five years preceding the main studied period (2011–2016). Sixty patients (lot A) were randomly selected who had already been admitted for dermatological reasons in previous years and who, at the time of their first admission, did not have an established diagnosis of diabetes. In other words, these were patients who developed and/or were diagnosed with diabetes in the time between their first and second admissions to our dermatology unit. At baseline (T1), baseline demographic parameters such as sex and urban/rural residency distribution did not differ significantly between the two studied sub-cohorts (Table 4).

**Figure 2:**
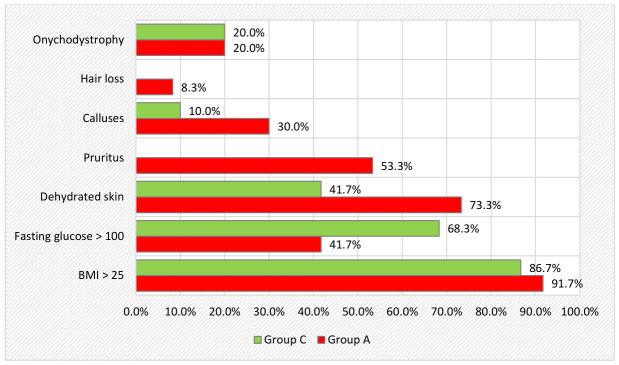
Initial assessments for Group A and Group C.

These patients were mostly male (61.7%), from both urban and rural backgrounds (53.3% vs. 46.7%), aged 40 to 80 years old (mean age 67). On their first admission, the vast majority (91.7%) were overweight or clinically obese, with body mass indices between 25 and 45 kg/m^2^. Fasting glucose levels were within the normal range (<100 mg/dL) in 58.3% of cases, suggesting the underlying presence of undiagnosed prediabetes or diabetes in at least some patients. The most common dermatological signs and symptoms notes were dehydrated skin (73.3%), pruritus (53.3%), calluses and hyperkeratosis (30%), onychomycoses and nail dystrophy (20%), and hair loss (8.33%).The baseline clinical and metabolic profiles obtained during the first hospital admission (2011–2016) are presented in Table 5, with the main pre-diagnostic cutaneous signs for Group A and Group C comparatively highlighted in Figure 2.

The control group (lot C) was made up of 60 patients with a mean age of 67, who were mostly men (63.3%) and mostly urban residents (56.7%) without established diabetes during either hospitalization. On their first admission (during 2011–2016), 86.7% were overweight or clinically obese, and 68.3 % had elevated fasting glucose levels. The aforementioned dermatological manifestations were also less present in this group: there were 41.7% patients with dehydrated skin, 10% with calluses, 20% with onychomycoses, and none with hair loss. No significant differences were noted with regard to weight, glycemia, or abnormal skin features during the second admission (during 2017–2022).

The data comparison revealed that patients diagnosed with diabetes in the time between hospitalizations presented initially with a higher incidence of dermatological signs and symptoms.

The Kolmogorov–Smirnov test indicated a non-normal distribution for most variables (*p* < 0.05), except for blood glucose at T2 (*p* = 0.058). Accordingly, the data are presented as the median and interquartile range (IQR) for non-normally distributed variables, while the mean ± standard deviation is reported only for blood glucose at T2.

At the first admission, no significant differences were observed between groups regarding age, sex, place of residence, and initial BMI. Baseline blood glucose was significantly higher in Group C compared to Group A (*p* = 0.003). Skin changes such as dry skin (73.3% vs. 41.7%, *p* < 0.001), pruritus (53.3% vs. 0%, *p* < 0.001), and callus (30.0% vs. 10.0%, *p* = 0.006) were significantly more frequent in Group A.

At the second admission, no significant differences were found between groups in terms of BMI and blood glucose. Dry skin remained significantly more frequent in Group A (78.3% vs. 43.3%, *p* < 0.001), while pruritus was present only in Group A (28.3% vs. 0%, *p* < 0.001). Onychomycosis was more frequent in Group C (43.3% vs. 21.7%, *p* = 0.011).

In Group A, blood glucose increased significantly from T1 to T2 (*p* = 0.008), while pruritus decreased (*p* = 0.007). In Group C, the only significant change was an increase in the prevalence of onychomycosis (*p* < 0.001). Clinical findings, anthropometric metrics, and dermatological status recorded at the second admission interval (2017–2022) are thoroughly summarized in Table 6.

The internal longitudinal analysis tracking the progression of clinical and skin parameters from baseline to T2 within the diabetes-development group (Lot A) is detailed in Table 7.

Comparatively, the chronological changes in clinical signs and associated *p*-values evaluated via the McNemar test for the non-diabetic control group (Lot C) are displayed in Table 8.

In both Group A and Group C, blood glucose levels, BMI, and skin appearance at the first admission were found to be predisposing factors for the diagnosis of diabetes mellitus (confirmed at the second admission). Univariate analysis focusing on clinical characteristics at baseline identified three specific cutaneous findings—dry skin, pruritus, and callus—as significant predisposing risk factors for a future diabetes diagnosis (Table 9).

We have therefore identified three risk factors—dry skin, pruritus (itching), and callus—which we will include in a multivariate binary logistic regression model to investigate their combined effect on the risk of developing diabetes.

Univariate analysis at T1 identified three risk factors for developing diabetes: dry skin (OR = 5.93, 95% CI: 2.70–13.07, *p* < 0.001), pruritus (OR = 5.09, 95% CI: 2.23–11.65, *p* < 0.001), and callus (OR = 2.79, 95% CI: 1.10–7.04, *p* = 0.027). The multivariate logistic regression model confirmed the independent association of dry skin (OR = 4.70, 95% CI: 2.02–10.96, *p* < 0.001) and pruritus (OR = 3.41, 95% CI: 1.39–8.37, *p* = 0.007) with the risk of diabetes.

The final multivariate binary logistic regression model confirmed both dry skin (OR = 4.700) and pruritus (OR = 3.410) as robust, independent predictors of future type 2 diabetes development (Table 10).

The model, built using the Enter method, is statistically significant and identifies dry skin and pruritus as risk factors, as shown in the following table:

**Table 10:** Multivariate Analysis of the three identified risk factors.

	B	Sig.	OR	95% C.I. for OR	
				Lower	Upper
Dry skin	1.548	<0.001	4.700	2.015	10.962
Pruritus	1.227	0.007	3.410	1.389	8.374
Callus	1.033	0.054	2.810	0.981	8.052
Constant	−1.453	<0.001	0.234		

## 4. Discussion

This study reports on the incidence of dermatological conditions in diabetes patients seeking diagnosis and treatment in a large tertiary care unit in NE Romania over a multi-year period. The research is relevant considering that such data is scarce in the literature and textbook information does not fully reflect the realities of clinical practice, yet 30% to 90% of diabetes patients eventually develop skin conditions and complications, impacting overall health and quality of life [17].

Our retrospective analysis of hospital records from 2017 to 2022 led to the identification of 960 diabetes patients (57% over the age of 65, 51.8% men, 58.3% urban residents), of whom most had type 2 diabetes (96.3%). A key finding of our study is that most lesions were ulcerative (85.7%) and inflammatory (12.4%), without significant differences relative to sex, background, or type of diabetes and with substantial consistency across a 6-year period.

A classification of the lesions was attempted, but many types described in the literature were rare or absent (only 1.9% of cases). This is not to say that patients in our region did not experience these conditions, but that they likely sought medical assistance in primary and secondary care settings, or informally (self-treatment), or disregarded their skin issues. Even so, our study suggests that a revision of classifications still referenced in Romania may be opportune to more accurately reflect the actual incidence of dermatological conditions and complications in diabetes patients.

In this study, we investigated the potential role of certain cutaneous manifestations as early predictors for the development of type 2 diabetes mellitus (T2DM). Our analysis focused on two matched groups of patients who were initially free of diabetes, with Group A presenting suggestive skin changes at baseline and Group C (controls) lacking such manifestations. The primary objective was to determine whether the presence of specific dermatological findings at baseline could be associated with an increased risk of subsequent T2DM diagnosis.

At baseline, demographic and anthropometric characteristics such as age, sex, residence, and BMI did not differ significantly between the groups. This suggests that the observed differences in outcomes are unlikely to be confounded by these variables. However, fasting blood glucose was significantly higher in Group C compared to Group A at the first admission. This is consistent with previous evidence that elevated fasting glucose, even within the non-diabetic range, is associated with a higher risk of progression to diabetes [18,19].

Our most notable finding was that dry skin, pruritus, and callus were significantly more prevalent in Group A at baseline. The high prevalence of these conditions in individuals without diagnosed diabetes suggests that subtle metabolic or microvascular changes may occur long before hyperglycemia reaches diagnostic thresholds. Dry skin (xerosis) has been linked to impaired epidermal barrier function, autonomic neuropathy, and reduced sweat gland activity in prediabetic and early diabetic states [15]. Pruritus, in turn, may be the result of altered skin hydration, microangiopathy, and low-grade systemic inflammation, all of which have been reported in insulin resistance and prediabetes [14,20]. Callus formation, particularly in plantar areas, has been associated with altered plantar pressure distribution and subclinical neuropathy [21].

At the second admission (T2), dry skin remained significantly more common in Group A, and pruritus continued to be present exclusively in this group. Interestingly, onychomycosis was more frequent in Group C, which might be explained by prolonged exposure to hyperglycemia and associated immune dysfunction. Fungal nail infections are well-documented in established diabetes, reflecting compromised host defenses [16]. The observed increase in onychomycosis prevalence in Group C over time suggests that it may develop later in the course of metabolic deterioration, whereas conditions like pruritus and xerosis may appear earlier.

When examining changes within each group over time, Group A showed a significant increase in fasting glucose and a decline in pruritus prevalence. The reduction in pruritus could be due to the progression of peripheral neuropathy, which may reduce itch perception. In Group C, the most significant change was an increase in onychomycosis prevalence, reinforcing its association with later-stage hyperglycemia.

Risk factor analysis revealed that, at baseline, dry skin, pruritus, and callus were associated with significantly increased odds of developing diabetes. In the multivariate model, dry skin (OR 4.70) and pruritus (OR 3.41) remained independent predictors after adjusting for confounders. This finding aligns with previous research demonstrating that dermatological manifestations can serve as valuable clinical markers for underlying metabolic dysfunction [22].

Clinically, these results suggest that routine skin examination could be an important adjunct in diabetes risk assessment, especially in primary care and dermatology settings. Health professionals should be aware that persistent xerosis, unexplained pruritus, or recurrent callus formation may warrant further metabolic evaluation. Such an approach is inexpensive, non-invasive, and could enable earlier intervention in individuals at risk.

The pathophysiological mechanisms underlying these associations likely involve early microvascular damage, neuropathic changes, and systemic inflammatory responses that begin before hyperglycemia becomes overt. Several studies have documented that microangiopathy and neuropathy may precede the diagnosis of diabetes by years, manifesting as subtle skin and nerve changes [23,24].

Our results align with literature reports of lower limb ulcerations being a common complication in diabetes and partially related to bacterial superinfections [25]. The association between diabetes and various types of infections has already been demonstrated and systematically reported [26]. In our results, 18.6% of patients had venous ulcers, and 14.7% of these lesions were positive for *Staphylococcus aureus*, while *Beta-hemolytic Streptococci* were evidenced in another 1.5% of samples. This incidence of infections is lower than in other studies. For instance, in research conducted on 750 diabetes patients ten years ago, 47.5% had infected lesions; likewise, we found fewer cases of inflammatory lesions (12.4% vs. 20.7%) [15]. On the other hand, mycoses of the toenails were diagnosed in 21.7% of cases, which is consistent with another recent study that demonstrated predisposition to onychomycoses in diabetes patients [27].

Age is another aspect for which our results provide fresh insights. Relative to the overall average age of 61 of patients developing diabetic foot ulcers, ulcerations in our study were highly common both in older as well as younger patients, unlike in other retrospective studies [28].

A multitude of factors are involved in the development of lower limb ulcerations in patients with diabetes, mostly related to the underlying chronic complications of diabetes. Our results support this understanding: first, high glycated hemoglobin levels (>7%) were noted in almost all of the diabetes patients presenting with skin lesions (94.2%). Moreover, the most severe ulcerations and bacterial superinfections were diagnosed in patients with HbA1C levels in excess of 10%. Other similar associations between high HbA1C levels and unfavorable outcomes of skin ulcers have been reported in the literature [29].

The integration of skin examination into standard diabetes risk assessment protocols would require minimal additional resources yet could yield significant public health benefits. Given the global burden of T2DM and the high proportion of undiagnosed cases, especially in low- and middle-income countries, the inclusion of skin-based indicators in opportunistic and community-based screening programs could be transformative. Such an approach would be particularly valuable in settings where laboratory diagnostics are limited or cost-prohibitive. Furthermore, these visible cutaneous markers could serve as practical clinical complements to validated metabolic screening frameworks, such as the non-invasive Finnish Diabetes Risk Score (FINDRISC), which are widely utilized to predict long-term type 2 diabetes progression in the general population [30].

Beyond its immediate screening utility, the recognition of xerosis and pruritus as risk markers opens avenues for more personalized preventive strategies. For example, patients identified with these signs could be prioritized for lifestyle counseling, dietary modification, and, where appropriate, pharmacological interventions aimed at delaying or preventing the onset of diabetes. Importantly, structured preventive measures—such as intensive diet and physical activity modifications—have been demonstrated in large-scale clinical randomized controlled trials to significantly reduce diabetes incidence by up to 58% in high-risk populations. Long-term longitudinal follow-up data have additionally proven that early, target-driven lifestyle interventions exert a sustained, protective legacy effect, reducing overall cardiovascular and diabetes-related morbidity for more than two decades, underscoring the vital preventive potential of early clinical identification through non-invasive dermatological markers [31,32].

In a recent systematic review, diabetic foot ulcers were found to occur in approximately 18.6 million people worldwide on an annual basis, leading in many cases to amputations and even death [33]. On a positive note, another systematic review revealed that multidisciplinary management can reduce major complications in as many as 94% of cases [18]. One recommended approach for the management of diabetic foot ulcers is MADADORE, put forth in 2019 by Lazzarini et al., where M stands for metabolic/medication, A for assessment, D for debridement, A for antibiotics, D for dressing, O for offloading, R for referral, and E for education [34]. In short, the advice is to aim for metabolic control, to provide foot assessment and adequate care (debridement, antibiotics, dressing, pressure offloading, as necessary), to engage with other specialists, and to educate patients towards self-care.

The results reported and discussed should be considered in light of the study limitations. In the hospital database of admissions, diabetes complications are only signaled as present/absent via a single code (E11.71) and not described. This prevented us from being able to evaluate this undoubtedly relevant aspect; i.e., microvascular and neurological complications in diabetes have a direct bearing on the development of lower limb ulcerations. Similarly, certain details such as height and weight had to be manually extracted from the patients’ actual medical records, which is why BMI data were not available for the entire study cohort. In addition, while it is standard practice to measure glycated hemoglobin levels in diabetes patients, this parameter is generally not assessed in patients presenting for dermatological conditions unless they have a previously established diagnosis of diabetes. Last but not least, we should acknowledge that there may be inconsistencies in the note-taking practices of attending physicians when interviewing patients about their symptoms (e.g., pruritus).

Limitations: This study was conducted in a single tertiary dermatology clinic, which may influence the distribution and severity of the dermatological conditions observed. Patients referred to specialized dermatology services often present with more advanced, complex, or chronic skin lesions than those managed in primary care, endocrinology, podiatry, or nursing-led diabetic foot programs. As a result, ulcerative lesions may be overrepresented compared with community-based populations, while milder dermatoses may be underreported. Therefore, caution is warranted when generalizing our findings to other clinical settings and healthcare systems. Multi-center studies including primary care and multidisciplinary diabetic foot services would help confirm the external validity of these observations.

## 5. Conclusions

This longitudinal study was conducted with diabetes patients admitted for specialized diagnosis and treatment of skin lesions. The noted multi-year incidence of skin lesions in diabetes patients presenting in tertiary care settings differs from how dermatological conditions are generally organized in some teaching and reference materials, inviting further research and revision.

This study provides strong and consistent evidence that specific dermatological manifestations—most notably xerosis [14,19] (dry skin) and pruritus [15]—serve as independent predictors for the future development of type 2 diabetes mellitus (T2DM) in individuals without overt hyperglycemia at baseline. By leveraging a carefully matched cohort design, we were able to minimize confounding by demographic and anthropometric factors, thus reinforcing the robustness of our findings.

At the initial evaluation, these cutaneous signs were not only more prevalent in individuals who subsequently developed diabetes, but their association persisted after adjusting for potential confounders in multivariate logistic regression. The magnitude of these associations, with an almost fivefold increase in odds for xerosis [14,19] and a more than threefold increase for pruritus [15], approaches that observed for recognized metabolic risk factors such as impaired fasting glucose and obesity. This finding is especially noteworthy as it elevates the relevance of dermatological indicators to the level of established biochemical and anthropometric predictors in diabetes risk assessment.

From a mechanistic standpoint, the observed associations are biologically plausible and align with existing knowledge of the early pathophysiological events in glucose dysregulation. Microvascular impairment, even in its subclinical stages, can compromise nutrient and oxygen delivery to the skin, while peripheral autonomic neuropathy may disrupt sweat gland function, reducing skin hydration and altering barrier integrity. Concurrently, low-grade chronic inflammation—a hallmark of early metabolic syndrome—can further impair epidermal function and sensorial perception, resulting in symptoms such as pruritus [15]. These processes can manifest long before fasting plasma glucose or HbA1c levels surpass diagnostic thresholds, positioning skin changes as potential sentinel markers of underlying metabolic compromise.

The longitudinal component of this study provides additional depth to these findings. In Group A, fasting glucose levels increased significantly during follow-up, while the prevalence of pruritus [15] declined. This trend could reflect the neurodegenerative progression of diabetic neuropathy, wherein sensory loss diminishes the perception of itch. Conversely, in Group C, the significant rise in onychomycosis [4,21] prevalence likely represents a later manifestation of chronic hyperglycemia, characterized by immune dysregulation and an increased susceptibility to fungal infections. Together, these temporal patterns support the concept of a staged dermatological progression in the natural history of diabetes, from early functional changes to late structural and infectious complications.

Our results are consistent with, and extend, previous studies that have highlighted the skin as a target organ in diabetes. The prior literature has reported xerosis [14,19] in up to 50% of newly diagnosed diabetic patients and pruritus [15] in 10–25%, often preceding diagnosis by months or years. The novelty of our study lies in the prospective identification of these signs in patients who were initially non-diabetic and in demonstrating their predictive value for future disease development. This temporal link strengthens the argument for their integration into routine clinical assessment, particularly in at-risk populations.

The clinical implications of these findings are substantial. First, dermatological assessment offers a low-cost, non-invasive, and easily accessible screening modality that can be performed in both primary care and specialist settings without the need for laboratory infrastructure. Second, the visible and symptomatic nature of skin changes makes them tangible for patients, potentially facilitating earlier engagement with healthcare services. Third, these signs can complement existing diabetes risk scores, enhancing their predictive accuracy and enabling earlier stratification of individuals into targeted preventive interventions.

The integration of skin examination into standard diabetes risk assessment protocols would require minimal additional resources yet could yield significant public health benefits. Given the global burden [35,36] of T2DM and the high proportion of undiagnosed cases, especially in low- and middle-income countries, the inclusion of skin-based indicators in opportunistic and community-based screening programs could be transformative. Such an approach would be particularly valuable in settings where laboratory diagnostics are limited or cost-prohibitive.

Beyond its immediate screening utility, the recognition of xerosis [14,19] and pruritus [15] as risk markers opens avenues for more personalized preventive strategies. For example, patients identified with these signs could be prioritized for lifestyle counseling, dietary modification, and, where appropriate, pharmacological interventions aimed at delaying or preventing the onset of diabetes. Importantly, these preventive measures have been shown in large randomized controlled trials to reduce diabetes incidence by up to 58% in high-risk populations, underscoring the potential impact of early identification.

Looking ahead, further research should aim to validate these findings in larger and more diverse cohorts, encompassing different ethnicities, climates, and socioeconomic contexts. Mechanistic studies combining dermatological assessment with microcirculatory imaging, skin biopsies, and inflammatory biomarker profiling could elucidate the biological pathways linking skin health and metabolic regulation. Moreover, the integration of digital health tools, such as smartphone-based skin imaging and artificial intelligence algorithms, holds promise for expanding access to dermatological screening and enabling remote risk assessment.

In conclusion, our study demonstrates that xerosis [14,19] and pruritus [15], when present in individuals without overt diabetes, are not merely coincidental dermatological findings but significant, independent predictors of future T2DM. The routine incorporation of skin assessment into clinical practice offers a feasible, inexpensive, and impactful opportunity to enhance early detection and prevention strategies. As the prevalence of T2DM continues to rise globally, embracing innovative, accessible, and patient-centered approaches such as this will be essential to curbing the growing epidemic and improving long-term health outcomes.

### Future Research Directions

Future studies should aim to validate these findings in larger, multi-center cohorts and in clinical settings beyond dermatology, including primary care, endocrinology, and podiatry services. Prospective longitudinal studies would help clarify the temporal relationship between early skin changes and the progression to overt diabetes. Additionally, integrating dermatologic assessment into diabetes risk scores and exploring objective skin barrier or microcirculation biomarkers could enhance early detection strategies. Finally, evaluating the impact of patient education and preventive skin care programs on the incidence and severity of ulcerative complications represents an important avenue for clinical practice improvement.

## Figures and Tables

**Table 1:** General characteristics of patients with inflammatory, ulcerative, and other lesions.

Skin Lesions on Admission	Age ≤ 65		Age ≥ 65		Female		Male		Rural		Urban		Total	
	N	%	N	%	N	%	N	%	N	%	N	%	N	%
	Pearson Chi 0.018, *p* = 0.991				Pearson Chi 1.978, *p* = 0.372				Pearson Chi 3.356, *p* = 0.187					
Inflammatory	52	12.2%	67	12.5%	65	13.9%	54	11%	56	13.5%	63	11.6%	119	12.4%
Ulcerative	365	85.9%	458	85.6%	393	84.2%	430	87.2%	348	83.9%	475	87.2%	823	85.7%
Other	8	1.9%	10	1.9%	9	1.9%	9	1.8%	11	2.7%	7	1.3%	18	1.9%
Total	425	100%	535	100%	467	100%	493	100%	415	100%	545	100%	960	100%

**Table 2:** Incidence of studied lesions in patients with diabetes mellitus types 1 and 2.

Skin Lesions on Admission	Without DM		T1DM		T2DM		Total	
	N	%	N	%	N	%	N	%
	Pearson Chi 1.641, *p* = 0.801							
Inflammatory	10	14.7%	5	14.7%	104	12.1%	119	12.4%
Ulcerative	56	82.4%	29	85.3%	738	86%	823	85.7%
Other	2	2.9%	0	0%	16	1.9%	18	1.9%
Total	68	100%	34	100%	858	100%	960	100%

Note: Fisher’s exact test was used because expected cell counts <5. Note: Exact inference used due to small/zero cells—Fisher–Freeman–Halton exact test for R × C tables (or Fisher’s exact test for 2 × 2).

**Table 3:** Multi-annual incidence of inflammatory, ulcerative, and other lesions (2017–2022).

Skin Lesions on Admission	2017		2018		2019		2020		2021		2022		TOTAL	
	N	%	N	%	N	%	N	%	N	%	N	%	N	%
Inflammatory	16	9.1%	22	11.3%	19	9.8%	7	9.9%	28	22.8%	27	13.4%	119	12.4%
Ulcerative	154	87.5%	168	86.2%	174	89.7%	64	90.1%	93	75.6%	170	84.6%	823	85.7%
Other	6	3.4%	5	2.6%	1	0.5%	0	0%	2	1.6%	4	2.0%	18	1.9%
Total	176	100%	195	100%	194	100%	71	100%	123	100%	201	100%	960	100%

Pearson Chi-squared = 22.102/*p* = 0.015 Note: Fisher’s exact test was used because expected cell counts <5. Note: Exact inference used due to small/zero cells—Fisher–Freeman–Halton exact test for R × C tables (or Fisher’s exact test for 2 × 2).

**Table 4:** Characteristics at the first admission.

		Lot				Pearson Chi-Squared *p*-Value
		A		C		
		n	%	n	%	
Sex	male	37	61.7%	38	63.3%	0.850
	female	23	38.3%	22	36.7%	
Environment	urban	32	53.3%	34	56.7%	0.714
	rural	28	46.7%	26	43.3%	
Total		60	100.0%	60	100.0%	

Note: Fisher’s exact test was used because expected cell counts <5. Note: Exact inference used due to small/zero cells—Fisher–Freeman–Halton exact test for R × C tables (or Fisher’s exact test for 2 × 2).

**Table 5:** Initial assessments.

Initial Assessments		Lot				Pearson Chi-Squared *p*-Value
		A		C		
		n	%	n	%	
Initial BMI	>25	55	91.7%	52	86.7%	0.378
	≤25	5	8.3%	8	13.3%	
Initial blood glucose	>100	25	41.7%	41	68.3%	0.003
	≤100	35	58.3%	19	31.7%	
Initial dry skin	Present	44	73.3%	25	41.7%	<0.001
	Absent	16	26.7%	35	58.3%	
Initial pruritus	Present	32	53.3%	0	0.0%	<0.001
	Absent	28	46.7%	60	100.0%	
Initial callus	Present	18	30.0%	6	10.0%	0.006
	Absent	42	70.0%	54	90.0%	
Initial hair loss	Present	5	8.3%	0	0.0%	0.057
	Absent	55	91.7%	60	100.0%	
Initial onychomychosis	Present	12	20.0%	12	20.0%	1.000
	Absent	48	80.0%	48	80.0%	
Total		60	100.0%	60	100.0%	

Note: Fisher’s exact test was used because expected cell counts <5. Note: Exact inference used due to small/zero cells—Fisher–Freeman–Halton exact test for R × C tables (or Fisher’s exact test for 2 × 2).

**Table 6:** Assessments at T2.

Assessments at T2		Lot				Pearson Chi-Squared *p*-Value
		A		C		
		n	%	n	%	
BMI at T2	>25	58	96.7%	53	88.3%	0.163
	≤25	2	3.3%	7	11.7%	
Blood glucose at T2	>100	37	61.7%	43	71.7%	0.245
	≤100	23	38.3%	17	28.3%	
Dry skin at T2	Present	47	78.3%	26	43.3%	<0.001
	Absent	13	21.7%	34	56.7%	
Pruritus at T2	Present	17	28.3%	0	0.0%	<0.001
	Absent	43	71.7%	60	100.0%	
Callus at T2	Present	19	31.7%	7	11.7%	0.008
	Absent	41	68.3%	53	88.3%	
Hair loss at T2	Present	3	5.0%	0	0.0%	0.244
	Absent	57	95.0%	60	100.0%	
Onychomycosis at T2	Present	13	21.7%	26	43.3%	0.011
	Absent	47	78.3%	34	56.7%	
Total		60	100.0%	60	100.0%	

Note: Fisher’s exact test was used because expected cell counts <5. Note: Exact inference used due to small/zero cells—Fisher–Freeman–Halton exact test for R × C tables (or Fisher’s exact test for 2 × 2).

**Table 7:** Assessments for Group A: initial versus T2.

Lot A						McNemar Test (Paired Samples) *p*-Value
		Initial		Mom. T2		
		n	%	n	%	
Initial BMI	>25	55	91.7%	58	96.7%	0.375
	≤25	5	8.3%	2	3.3%	
Initial blood glucose	>100	25	41.7%	37	61.7%	0.008
	≤100	35	58.3%	23	38.3%	
Initial dry skin	Present	44	73.3%	47	78.3%	0.690
	Absent	16	26.7%	13	21.7%	
Initial pruritus	Present	32	53.3%	17	28.3%	0.007
	Absent	28	46.7%	43	71.7%	
Initial callus	Present	18	30.0%	19	31.7%	1.000
	Absent	42	70.0%	41	68.3%	
Initial hair loss	Present	5	8.3%	3	5.0%	0.687
	Absent	55	91.7%	57	95.0%	
Initial onychomychosis	Present	12	20.0%	13	21.7%	1.000
	Absent	48	80.0%	47	78.3%	
Total		60	100.0%	60	100.0%	

Note: Fisher’s exact test was used because expected cell counts <5. Note: Exact inference used due to small/zero cells—Fisher–Freeman–Halton exact test for R × C tables (or Fisher’s exact test for 2 × 2).

**Table 8:** Assessments for control group (C): initial versus T2.

Lot C						McNemar Test (Paired Samples) *p*-Value
		Initial		Mom. T2		
		n	%	n	%	
Initial BMI	>25	52	86.7%	53	88.3%	1.000
	≤25	8	13.3%	7	11.7%	
Initial blood glucose	>100	41	68.3%	43	71.7%	0.500
	≤100	19	31.7%	17	28.3%	
Initial dry skin	Present	25	41.7%	26	43.3%	1.000
	Absent	35	58.3%	34	56.7%	
Initial pruritus	Present	0	0.0%	0	0.0%	-
	Absent	60	100.0%	60	100.0%	
Initial callus	Present	6	10.0%	7	11.7%	1.000
	Absent	54	90.0%	53	88.3%	
Initial hair loss	Present	0	0.0%	0	0.0%	-
	Absent	60	100.0%	60	100.0%	
Initial onychomycosis	Present	12	20.0%	26	43.3%	<0.001
	Absent	48	80.0%	34	56.7%	
Total		60	100.0%	60	100.0%	

Note: Fisher’s exact test was used because expected cell counts <5. Note: Exact inference used due to small/zero cells—Fisher–Freeman–Halton exact test for R × C tables (or Fisher’s exact test for 2 × 2).

**Table 9:** Analysis of risk factors: lot A vs. lot C.

Initial Assessments		Lot				Pearson Chi-Squared *p*-Value	OR (95% CI)
		A		C			
		n	%	n	%		
Sex	Male	37	61.7%	35	58.3%	0.709	
	Female	23	38.3%	25	41.7%		
Initial BMI	>25	55	91.7%	52	86.7%	0.378	
	≤25	5	8.3%	8	13.3%		
Initial blood glucose	>100	25	41.7%	30	50.0%	0.360	
	≤100	35	58.3%	30	50.0%		
Initial dry skin	Present	44	73.3%	19	31.7%	<0.001	5.934 (2.695 ÷ 13.069)
	Absent	16	26.7%	41	68.3%		
Initial pruritus	Present	32	53.3%	11	18.3%	<0.001	5.091 (2.225 ÷ 11.647)
	Absent	28	46.7%	49	81.7%		
Initial callus	Present	18	30.0%	8	13.3%	0.027	2.786 (1.103 ÷ 7.038)
	Absent	42	70.0%	52	86.7%		
Initial hair loss	Present	5	8.3%	3	5.0%	0.717	
	Absent	55	91.7%	57	95.0%		
Initial onychomychosis	Present	12	20.0%	11	18.3%	0.817	
	Absent	48	80.0%	49	81.7%		
Total		60	100.0%	60	100.0%		

## Data Availability

The data presented in this study are available on request from the corresponding author.

## References

[1] ElSayed N.A., Aleppo G., Aroda V.R., Bannuru R.R., Brown F.M., Bruemmer D., Collins B.S., Cusi K., Das S.R., Gibbons C.H. (2023). Introduction and methodology: Standards of Care in Diabetes—2023. Diabetes Care.

[2] Dack C., Ross J., Stevenson F., Pal K., Gubert E., Michie S., Yardley L., Barnard M., May C., Farmer A. (2019). A digital self-management intervention for adults with type 2 diabetes. Internet Interv..

[3] Park H.Y., Kim J.H., Jung M., Chung C.H. (2011). A longstanding hyperglycaemic condition impairs skin barrier ageing process. Exp. Dermatol..

[4] Trovato L., Calvo M., De Pasquale R., Scalia G., Oliveri S. (2022). Prevalence of Onychomycosis in Diabetic Patients. J. Fungi.

[5] de Macedo G.M., Nunes S., Barreto T. (2016). Skin disorders in diabetes mellitus. Diabetol. Metab. Syndr..

[6] Armstrong D.G., Boulton A.J.M., Bus S.A. (2022). Diabetic foot disease: Pathophysiology, epidemiology, and prevention. N. Engl. J. Med..

[7] Lee M.K., Lee D.Y., Ahn H.Y., Park C.Y. (2021). Tailored mobile coaching for diabetes. JMIR Mhealth Uhealth.

[8] Dening J., Islam S.M.S., George E., Maddison R. (2020). Web-based dietary interventions in diabetes. J. Med. Internet Res..

[9] Smith A.G., Lessard M., Reyna S., Doudova M., Singleton J.R. (2014). Diagnostic utility of Sudoscan. J. Diabetes Complicat..

[10] Gheucă-Solovăstru L., Țiplica G.S., Diaconu D., Hărăț-Batog A., Vâță D., Ioan B. (2010). Ethical premises in dermatology. Rev. Rom. Bioet..

[11] Pop-Busui R., Boulton A.J., Feldman E.L., Bril V., Freeman R., Malik R.A., Sosenko J.M., Ziegler D. (2017). Diabetic Neuropathy: ADA Position Statement. Diabetes Care.

[12] Freeman R. (2009). Differential diagnosis of neuropathy in diabetes. Curr. Diab. Rep..

[13] Piérard G.E., Seité S., Hermanns-Lê T., Delvenne P., Scheen A., Piérard-Franchimont C. (2013). The skin landscape in diabetes mellitus. Focus on dermocosmetic management. Clin. Cosmet. Investig. Dermatol..

[14] Lima A.L., Illing T., Schliemann S., Elsner P. (2017). Cutaneous manifestations of diabetes mellitus: A review. Am. J. Clin. Dermatol..

[15] Yosipovitch G., Goon A.T., Wee J., Chan Y.H., Zucker I., Goh C.L. (2009). Itch characteristics in Asian patients with diabetes mellitus. J. Eur. Acad. Dermatol. Venereol..

[16] Demirseren D.D., Emre S., Akoglu G., Arpacı D., Arman A., Metin A., Cakır B. (2014). Relationship between skin diseases and extracutaneous complications of diabetes mellitus: Clinical analysis of 750 patients. Am. J. Clin. Dermatol..

[17] American Diabetes Association (2024). Standards of Medical Care in Diabetes—2024. Diabetes Care.

[18] Tabák A.G., Herder C., Rathmann W., Brunner E.J., Kivimäki M. (2012). Prediabetes: A high-risk state for diabetes development. Lancet.

[19] Kinkelin I., Bröcker E.B., Goebeler M. (2000). Xerosis cutis: Frequency, clinical manifestation and therapeutic treatment in diabetes mellitus. Hautarzt.

[20] Bus S.A., Maas M., Michels R.P., Levi M. (2002). Role of intrinsic muscle atrophy in the etiology of claw toe deformity in diabetic neuropathy. Diabetes Care.

[21] Gupta A.K., Konnikov N., MacDonald P., Rich P. (1998). Prevalence and epidemiology of toenail onychomycosis in diabetic subjects: A multicentre survey. Br. J. Dermatol..

[22] Edwards J.L., Vincent A.M., Cheng H.T., Feldman E.L. (2008). Diabetic neuropathy: Mechanisms to management. Pharmacol. Ther..

[23] Malik R.A. (2014). Early detection of neuropathy in diabetes. BMJ.

[24] Bus S.A., van Deursen R.W., Armstrong D.G., Lewis J.E., Caravaggi C.F., Cavanagh P.R. (2016). International Working Group on the Diabetic Foot. Footwear and offloading interventions to prevent and heal foot ulcers and reduce plantar pressure in patients with diabetes: A systematic review. Diabetes Metab. Res. Rev..

[25] Abu-Ashour W., Twells L., Valcour J., Randell A., Donnan J., Howse P., Gamble J.M. (2017). The association between diabetes mellitus and incident infections: A systematic review and meta-analysis of observational studies. BMJ Open Diabetes Res. Care.

[26] Pop-Busui R., Cleary P.A., Braffett B.H., Martin C.L., Herman W.H., Low P.A., Lima J.A.C., Bluemke D.A. (2013). Association between cardiovascular autonomic neuropathy and left ventricular dysfunction: DCCT/EDIC study. J. Am. Coll. Cardiol..

[27] Cernea S. (2017). Clasificarea diabetului zaharat şi modelul etiopatogenetic general. Patogeneza Diabetului Zaharat.

[28] Rosinha P., Saraiva M., Ferreira L., Garrido S., Carvalho A., Freitas C., Amaral C., Costa L., Loureiro L., Carvalho R. (2022). A Retrospective Cohort Study on Diabetic Foot Disease: Ascertainment of Ulcer Locations by Age Group. Cureus.

[29] Tong K.P.S., Green S.J., Ortiz J., Wu S.C. (2022). Association between hemoglobin A1c, Vitamin C, and microbiome in diabetic foot ulcers and intact skin: A cross-sectional study. Health Sci. Rep..

[30] Lindström J., Tuomilehto J. (2003). The diabetes risk score: A practical tool to predict type 2 diabetes risk. Diabetes Care.

[31] Knowler W.C., Barrett-Connor E., Fowler S.E., Hamman R.F., Lachin J.M., Walker E.A., Nathan D.M., Watson P.G., Mendoza J.T., Smith K.A. (2002). Reduction in the incidence of type 2 diabetes with lifestyle intervention or metformin. N. Engl. J. Med..

[32] Li G., Zhang P., Wang J., Gregg E.W., Yang W., Gong Q., Li H., Li H., Jiang Y., An Y. (2008). The long-term effect of lifestyle interventions to prevent diabetes in the China Da Qing Diabetes Prevention Study: A 20-year follow-up study. Lancet.

[33] Armstrong D.G., Tan T.W., Boulton A.J.M., Bus S.A. (2023). Diabetic Foot Ulcers: A Review. JAMA.

[34] Lazzarini P.A., Fernando M.E., Van Netton J.J. (2019). Diabetic foot ulcers: Is remission a realistic goal?. Endocrinol. Today.

[35] Cho N.H., Shaw J.E., Karuranga S., Huang Y., da Rocha Fernandes J.D., Ohlrogge A.W., Malanda B.I.D.F. (2018). IDF Diabetes Atlas: Global estimates of diabetes prevalence for 2017 and projections for 2045. Diabetes Res. Clin. Pract..

[36] Forouhi N.G., Wareham N.J. (2014). Epidemiology of diabetes. Medicine.

